# Prevalence of *Borrelia burgdorferi* and *Anaplasma phagocytophilum* in Ixodid Ticks from Southeastern Ukraine

**DOI:** 10.1089/vbz.2020.2716

**Published:** 2021-03-25

**Authors:** Nadia Kovryha, Ala Tsyhankova, Olena Zelenuchina, Olexandr Mashchak, Roman Terekhov, Artem S. Rogovskyy

**Affiliations:** ^1^ The Zaporizhzhya Oblast Laboratory Center, the Ministry of Health of Ukraine, Zaporizhzhya, Ukraine.; ^2^ Department of Veterinary Pathobiology, College of Veterinary Medicine and Biomedical Sciences, Texas A&M University, College Station, Texas, USA.

**Keywords:** ticks, *Anaplasma phagocytophilum*, *Borrelia burgdorferi* s. l, *Ehrlichia chaffeensis*, Lyme borreliosis, Ukraine

## Abstract

***Objectives:*** Tick-borne diseases have emerged as an increasing medical problem in the world. Being the most prevalent ixodid ticks in Europe, *Ixodes ricinus* and *Dermacentor reticulatus* are responsible for transmission of numerous zoonotic pathogens (*e.g*., human granulocytic anaplasmosis and Lyme borreliosis). Despite their public health significance, studies on the prevalence of tick-borne agents are scare for Eastern Europe. The objective of this study was to examine the prevalence of *Anaplasma phagocytophilum*, *Ehrlichia chaffeensis*, and *Borrelia burgdorferi* sensu lato (*B. burgdorferi* s. l.) in ixodid ticks from Southeastern Ukraine.

***Methods:*** Over a 5-year period (2014–2018), 358 questing and 389 engorged ixodid ticks were collected from Southeastern Ukraine (Zaporizhzhya region). The ticks were identified as *Dermacentor marginatus*, *D. reticulatus*, *I. ricinus*, and *Rhipicephalus rossicus*. Nucleic acid samples extracted from tick pools were subjected to RT-PCR analyses for *A. phagocytophilum*, *E. chaffeensis*, and *B. burgdorferi* s. l.

***Results:*** The examined ixodid ticks tested negative for the aforementioned pathogens with the exception of *I. ricinus* ticks. For questing *I. ricinus* ticks, minimum infection rates of *A. phagocytophilum* and *B. burgdorferi* s. l. were, respectively, 4.2–7.7% and 8.6–12.7%.

***Conclusions:*** These findings will be valuable for medical and veterinary practitioners when risks associated with tick-borne diseases are assessed for southeastern regions of Ukraine.

## Introduction

To date, vector-borne diseases represent a health problem of increasing significance in the world. Ticks are the leading vectors of human and animal vector-borne diseases (Boulanger et al. 2019). In Europe, including Ukraine, *Ixodes ricinus* (Linnaeus, 1758) and *Dermacentor reticulatus* (Fabricius, 1794) are the most prevalent ixodid ticks (Acari: Ixodidae) (Akimov and Nebogatkin 1997, Rubel et al. 2016). *I. ricinus* ticks are the competent vector for such zoonotic agents as *Anaplasma phagocytophilum* and *Borrelia burgdorferi* sensu lato (*B. burgdorferi* s. l.) among others (Boulanger et al. 2019). In addition to being the proven main vector for some of *Babesia* spp. and *Rickettsia* spp., *D. reticulatus* ticks can also harbor other zoonotic agents (*e.g*., *A. phagocytophilum*) (Rubel et al. 2016). Despite the medical importance of the tick-borne diseases (Jahfari et al. 2014, Boulanger et al. 2019, Riccardi et al. 2019, Vandekerckhove et al. 2019, Rogovskyy et al. 2020, Yurchenko et al. 2020), very few studies have examined the prevalence of the aforementioned pathogens in Eastern Europe, specifically Ukraine. Currently, there are no data on the prevalence of zoonotic agents in ticks from any southern or eastern parts of Ukraine. Therefore, the objective of this study was to examine ixodid ticks collected from Southeastern Ukraine for the presence of *A. phagocytophilum*, *Ehrlichia chaffeensis*, and *B. burgdorferi* s. l.

## Materials and Methods

A total of 358 questing ixodid ticks were collected through flagging over a 5-year period (2014–2018) at several sites of Zaporizhzhya oblast (region): the City of Berdiansk, Cosmic microdistrict, Khortytsia island, Ridge Canal (Domaha), Velykyi Luh, Zaporizkyi district, Vilnianskyi district, and the City of Zaporizhzhya. Based on their morphology (Filippova 1977), the questing ticks were identified as *Dermacentor marginatus* (10 females), *D. reticulatus* (11 females), *I. ricinus* (220 females, 35 males, and 63 nymphs; in a total of 318 ticks), and *Rhipicephalus rossicus* (19 females). During the 5-year period, a total of 389 engorged ticks of the respective tick species were obtained from the Zaporizhzhya Oblast Laboratory Center of the Ministry of Health of Ukraine. Of the 389 ticks, 237 engorged ticks, which included 1 *D. marginatus* tick (1 female), 6 *D. reticulatus* ticks (5 females and 1 male), 209 *I. ricinus* ticks (123 females, 14 males, and 72 nymphs), and 21 *Rh. rossicus* ticks (13 females, 2 males, and 6 nymphs), were collected from humans. The other 152 engorged ticks, which comprised 8 *D. marginatus* ticks (8 females), 16 *D. reticulatus* ticks (10 females, 1 male, and 5 nymphs), 110 *I. ricinus* ticks (94 females, 11 males, and 5 nymphs), and 18 *Rh. rossicus* ticks (14 females and 4 males), were originated from other animals (Table 1). Of the 152 ticks, 8 *D. marginatus* ticks were collected from a dog (2 females) and 4 cattle (6 females); 16 *D. reticulatus* ticks were collected from 3 dogs (2 females and 1 male) and 5 cattle (8 females and 5 nymphs); 110 *I. ricinus* ticks were collected from 20 dogs (77 females, 11 males, and 5 nymphs), 3 cattle (15 females), and a cat (2 females); and 18 *Rh. rossicus* ticks were collected from 2 dogs (1 female and 4 males) and 3 cattle (13 females). The collected ticks were immersed in 70% ethanol and stored at 4°C until analyses.

**Table 1.** The Prevalence of *Anaplasma phagocytophilum*, *Borrelia burgdorferi* Sensu Lato, and *Ehrlichia chaffeensis* in Ixodid Ticks Collected from Vegetation, Humans, and Other Animals

Pathogens	Pools of ticks collected from												Total
	Vegetation			Humans			Other animals			Total by tick stage			
	F	M	N	F	M	N	F	M	N	F	M	N	
*Ixodes ricinus*													
*A. phagocytophilum*	10/32 (172)^a^	2/4 (26)	2/7 (48)	5/62 (83)	0/4 (4)	2/30 (43)	2/13 (64)	0/2 (11)	0/1 (1)	17/107 (319)	2/10 (41)	4/38 (92)	23/155 (452)
*B. burgdorferi* s. l.	27/39 (220)	3/5 (35)	8/11 (63)	21/102 (123)	0/14 (14)	15/58 (72)	6/20 (94)	0/2 (11)	2/3 (5)	54/161 (437)	3/21 (60)	25/72 (140)	82/254 (637)
*E. chaffeensis*	0/32 (172)	0/4 (26)	0/7 (48)	0/62 (83)	0/4 (4)	0/30 (43)	0/13 (64)	0/2 (11)	0/1 (1)	0/107 (319)	0/10 (41)	0/38 (92)	0/155 (452)
*Dermacentor marginatus*													
*A. phagocytophilum*	0/5 (10)	n/a	n/a	0/1 (1)	n/a	n/a	0/5 (8)	n/a	n/a	0/11 (19)	n/a	n/a	0/11 (19)
*E. chaffeensis*	0/5 (10)	n/a	n/a	0/1 (1)	n/a	n/a	0/5 (8)	n/a	n/a	0/11 (19)	n/a	n/a	0/11 (19)
*Dermacentor reticulatus*													
*A. phagocytophilum*	0/7 (11)	n/a	n/a	0/5 (5)	0/1 (1)	n/a	0/5 (10)	0/1 (1)	0/2 (5)	0/17 (26)	0/2 (2)	0/2 (5)	0/21 (33)
*E. chaffeensis*	0/7 (11)	n/a	n/a	0/5 (5)	0/1 (1)	n/a	0/5 (10)	0/1 (1)	0/2 (5)	0/17 (26)	0/2 (2)	0/2 (5)	0/21 (33)
*Rhipicephalus rossicus*													
*A. phagocytophilum*	0/5 (19)	n/a	n/a	0/12 (13)	0/2 (2)	0/5 (6)	0/4 (14)	0/1 (4)	n/a	0/21 (46)	0/3 (6)	0/5 (6)	0/29 (58)
*E. chaffeensis*	0/5 (19)	n/a	n/a	0/12 (13)	0/2 (2)	0/5 (6)	0/4 (14)	0/1 (4)	n/a	0/21 (46)	0/3 (6)	0/5 (6)	0/29 (58)

^a^ Values correspond to number of PCR-positive tick pools/total numbers of tick pools tested. In the parentheses are indicated the total number of individual ticks tested per a total number of pools.

F, female; M, male; N, nymphal ticks; n/a, nonapplicable.

Nucleic acid (DNA and RNA) was extracted from pooled ticks (1–10 ticks per pool). For that, commercially available nucleic acid extractions kits, “RIBO-Prep” (AmpliPrime) and “RealBest Extraction 100” (Vector-Best) were used. DNA isolation control was also used according to the manufacturers' instructions. All the samples were then subjected to a multiplex RT-PCR. The pools were tested through “AmpliSens TBEV, *B. burgdorferi* s. l., *A. phagocytophilum, E. chaffeensis*/*E. muris-F*” and/or “RealBest DNA *Borrelia burgdorferi* s. l.” (Central Research Institute for Epidemiology) by using the iCycler iQ5 thermocycler (BioRad Laboratories) according to the manufacturers' instructions. Pools of *I. ricinus*, *D. marginatus*, *D. reticulatus*, or *Rh. rossicus* ticks were examined for the presence of *A. phagocytophilum* and *E. chaffeensis*. *I. ricinus* ticks were also analyzed for *B. burgdorferi* s. l. The numbers of ticks and their pools tested are provided in Table 1. Where applicable, minimum infection rates (%) of *A. phagocytophilum* and *B. burgdorferi* s. l. were calculated for *I. ricinus* ticks with the assumption that only one tick in each positive pool was infected (no. of ticks tested/minimal no. infected; Table 2). Importantly, the internal positive and negative controls provided by the kits were included in each PCR reaction. No approval from institutional ethics committee was required for this study.

**Table 2.** The Minimum Infection Rates of *Anaplasma phagocytophilum* and *Borrelia burgdorferi* Sensu Lato in *Ixodes ricinus* Ticks

Pathogens	Pools of ticks collected from											
	Vegetation			Humans			Other animals			Total by tick stage		
	F	M	N	F	M	N	F	M	N	F	M	N
*A. phagocytophilum*	5.8^a^	7.7	4.2	6.0	n/a	4.7	3.1	n/a	n/a	5.3	4.9	4.3
*B. burgdorferi* s. l.	12.3	8.6	12.7	17.1	n/a	20.8	6.4	n/a	40.0	12.3	5.0	17.9

^a^ Values correspond to minimum infection rates (%), which were calculated with the assumption that only one tick in each positive pool was infected (no. of ticks tested/minimal no. infected).

## Results

The PCR results showed that all the analyzed pools of *D. marginatus* (*n* = 11), *D. reticulatus* (*n* = 21), and *Rh. rossicus* (*n* = 29) ticks were negative for *A. phagocytophilum* and *E. chaffeensis* (Table 1). Furthermore, all the pools of *I. ricinus* ticks (*n* = 155) tested negative for *E. chaffeensis* as well. In contrast, a total of 14.8% (23 PCR-positive pools out of 155 pools tested; 23/155) pools of questing and engorged *I. ricinus* adults were positive for *A. phagocytophilum*. The highest proportions of *A. phagocytophilum*-positive pools were detected for questing *I. ricinus* adults (33.3%; 12/36) followed by 13.3% (2/15) and 7.6% (5/66) of engorged *I. ricinus* adults collected from animals and humans, respectively. In addition, a total of 4 pools of questing (2/7) and engorged *I. ricinus* nymphs (2/31) were PCR-positive for *A. phagocytophilum* (Table 1). The calculated minimum infection rates of *I. ricinus* ticks for *A. phagocytophilum* are provided in Table 2.

Besides the two tick-borne pathogens tested, some pools of *I. ricinus* adults and nymphs were also examined for the presence of *B. burgdorferi* s. l. The results demonstrated that a total of 32.3% of pools of both questing and engorged *I. ricinus* ticks carried *B. burgdorferi* s. l. DNA (Table 1). The highest percentage of positive pools of *I. ricinus* ticks were observed for questing nymphs (72.7%; 8/11) and adults (68.2%; 30/44). Furthermore, 18.1% pools of engorged adults (21/116) and 25.9% pools of engorged nymphs (15/58) collected from humans were PCR-positive for *B. burgdorferi* s. l. Finally, 27.3% (6/22) and 66.7% (2/3) pools of engorged adults and nymphs of *I. ricinus* collected from animals tested positive for *B. burgdorferi* s. l. DNA (Table 1). The minimum infection rates of questing *I. ricinus* ticks for *B. burgdorferi* s. l. were 12.3%, 8.6%, and 12.7% for females, males, and nymphs, respectively (Table 2).

## Discussion

This study demonstrated that, in Southeastern Ukraine (Zaporizhzhya region), the minimum infection rates of *A. phagocytophilum* for *I. ricinus* ticks varied from 3.1% for engorged females and 4.2% for questing nymphs to 6.0% for engorged females and 7.7% for questing males (Table 2). These rates were comparable with the infection rates of *A. phagocytophilum*, which were previously recorded for individually tested questing *I. ricinus* ticks collected in Eastern Ukraine (3.6%), Central Ukraine (2.7–5.2%), and Northern Ukraine (0.4%) (Movila et al. 2009, Didyk et al. 2017, Rogovskyy et al. 2017, 2018, 2019). Similar to Ukraine, low infection rates of *A. phagocytophilum* were reported in questing *I. ricinus* ticks from neighboring countries such as Belarus (4.2%), Lithuania (2.9%), Moldova (5.1–9.0%), Poland (10.7%), Russia (3.1%), and Slovakia (5.5%) (Grzeszczuk et al. 2004, Koci et al. 2007, Radzijevskaja et al. 2008, Movila et al. 2009, Pangracova et al. 2013, Reye et al. 2013, Kiewra et al. 2014, Livanova et al. 2018).

This investigation did not identify *A. phagocytophilum* DNA in *D. marginatus* and *D. reticulatus* ticks. The lack of detection could be explained by low numbers of ticks analyzed and an overall low infection rate of this pathogen in *Dermacentor* ticks reported for Ukraine in the past (Movila et al. 2009, Didyk et al. 2017, Rogovskyy et al. 2017, 2018, 2019). Recent study showed that only 1.0% of questing *D. reticulatus* ticks (a total of 98 individual ticks analyzed; *n* = 98) from Central Ukraine (Kyiv) carried *A. phagocytophilum* DNA (Rogovskyy et al. 2018). Moreover, all individually analyzed ticks of *D. reticulatus* collected from Northern Ukraine (*n* = 100) and neighboring Belarus (*n* = 164) were consistently PCR-negative for *A. phagocytophilum* (Reye et al. 2013, Rogovskyy et al. 2019). In addition to *A. phagocytophilum*, the ticks were also examined for another member of the *Anaplasmataceae* family, *E. chaffeensis*, the causative agent of human monocytic ehrlichiosis, which is extremely rare in Europe (Yabsley 2010). Expectedly, this study did not detect *E. chaffeensis* DNA in the examined ticks.

*Rh. rossicus* (Jakimov et Kohl-Jakimova, 1911) and infection rates of various pathogens for this vector are highly understudied. This tick species is known to have a vectoral role for only a few pathogens (*Francisella tularensis*, Crimean-Congo hemorrhagic fever virus, and West Nile virus) and, for most European countries, *Rh. rossicus* (Jakimov et Kohl-Jakimova, 1911) is considered an alien tick (Mihalca et al. 2015). *Rh. rossicus* is indigenous to Ukraine. Recently, the habitat of *Rh. rossicus* was considerably expanded from the south to the north of Ukraine (Mihalca et al. 2015). This study demonstrated that all the examined *Rh. rossicus* ticks tested negative.

Although Lyme borreliosis (LB) has been steadily on the rise in Ukraine (Carriveau et al. 2019, Rogovskyy et al. 2020), very few studies have investigated prevalence of *B. burgdorferi* s. l. in ixodid ticks from this country (Movila et al. 2009, Didyk et al. 2017, Rogovskyy et al. 2017, 2018, 2019). Furthermore, the published epidemiological data were generated only for Central and Western Ukraine (Movila et al. 2009, Didyk et al. 2017, Rogovskyy et al. 2017, 2018, 2019, Weiner et al. 2018). Thus, this study examined the prevalence of *B. burgdorferi* s. l. in *I. ricinus* ticks from Southeastern Ukraine. The results demonstrated that the minimum infection rates of *B. burgdorferi* s. l. in questing *I. ricinus* ticks were 12.3%, 8.6%, and 12.7% for females, males, and nymphs, respectively (Table 2). Overall, these rates are consistent with the findings of previous studies, where 4.4% and 5.2–13.5% of individually analyzed ticks from Western (Ternopyl region) and Central (Kyiv and the CEZ) Ukraine were found to carry *B. burgdorferi* s. l. DNA (Didyk et al. 2017, Rogovskyy et al. 2018, 2019, Weiner et al. 2018).

## Conclusions

This study is the first to investigate the prevalence of *A. phagocytophilum*, *B. burgdorferi*, and *E. chaffeensis* in ixodid ticks collected from Southeastern Ukraine. Pools of *I. ricinus*, *D. marginatus*, *D. reticulatus*, or *Rh. rossicus* were examined for the presence of *A. phagocytophilum* and *E. chaffeensis* by PCR. The data demonstrated that all the ticks were consistently negative for the two pathogens with the exception of *I. ricinus* ticks, which tested positive for *A. phagocytophilum*. Moreover, *I. ricinus* ticks were also analyzed for *B. burgdorferi* s. l. The result demonstrated that 12.3%, 8.6%, and 12.7% of females, males, and nymphs, respectively, tested positive for *B. burgdorferi* s. l. The newly provided data should be highly valuable for both medical and veterinary practitioners as well as epidemiologists when assessing the risks associated with tick-borne diseases for Southeastern Ukraine.
